# The Progression Related Gene *RAB42* Affects the Prognosis of Glioblastoma Patients

**DOI:** 10.3390/brainsci12060767

**Published:** 2022-06-11

**Authors:** Liwei Sun, Tao Yan, Bing Yang

**Affiliations:** 1 Department of Oncology, Tianjin Huanhu Hospital, Tianjin Key Laboratory of Cerebral Vascular and Neurodegenerative Disease, Tianjin Neurosurgical Institute, Tianjin 300350, China; daerduoni2005@163.com; 2 Department of Pharmacy, Tianjin Huanhu Hospital, Tianjin Key Laboratory of Cerebral Vascular and Neurodegenerative Disease, Tianjin Neurosurgical Institute, Tianjin 300350, China; 3 Department of Cell Biology, College of Basic Medical Sciences, Tianjin Medical University, Tianjin 300070, China; bingyang@tmu.edu.cn

**Keywords:** glioblastoma, *RAB42*, The Cancer Genome Atlas, prognostic marker

## Abstract

Background: Glioblastoma (GBM) represents the most malignant glioma among astrocytomas and is a lethal form of brain cancer. Many RAB genes are involved in different cancers but *RAB42* (Ras-associated binding 42) is seldom studied in GBM. Our study aimed to explore the role of *RAB42* expression in the development and prognosis of GBM. Methods: All GBM patient data were obtained from The Cancer Genome Atlas (TCGA) and Chinese Glioma Genome Atlas (CGGA) databases. The relevance of *RAB42* expression to the clinicopathologic characteristics of GBM patients was analyzed. The overall survival (OS) significance was determined using log-rank. Significantly enriched KEGG pathways were screened using gene set enrichment analysis (GSEA). Results: High expression of *RAB42* was observed in GBM specimens compared with normal samples, which was also verified in cell lines and tissue samples. Elevated *RAB42* expression was correlated with higher GBM histological grade. The prognosis of GBM patients with high *RAB42* expression was worse than those with lower *RAB42*. A total of 35 pathways, such as the P53 pathway, were significantly activated in highly *RAB42*-expressed GBM samples. Conclusions: High *RAB42* expression is related to the development of GBM, and *RAB42* is a probable prognostic marker for GBM.

## 1. Introduction

Glioblastomas (GBM), also named WHO grade IV astrocytomas [1], are intrinsic brain tumors [2]. In addition, GBM is the most malignant glioma among astrocytomas and is a lethal form of brain cancer [3]. The median overall survival (OS) of GBM was just 10–20 months with radio- and chemotherapy in [4,5,6]. Unlike some other cancers, therapies for and research into GBM are facing more challenges due to limited opportunities for reoperation, limited tumor locations, limited sample amounts, and so on [7,8,9]. Not only that, there is a significantly heterogeneous inter- and intra-tumor genome in GBMs [8], which is a problem in various kinds of cancers. However, based on the background of a high probability of 5-year recurrence for GBM [10], not only do some estimates suggest that magnetic resonance imaging (MRI) pseudoprogression rates are close to 20% [11] but also significant differences could be observed in the survival outcomes of GBM patients with conventional prognostic factors [12]. Accordingly, it is imperative to explore novel targeted progression and prognostic markers for GBM patients.

Ras-associated binding (RAB) proteins constitute the largest family in the RAS superfamily of small GTPases, including over 60 identified members in humans [13,14]. *RAB42* is a member of the RAB family. Aberrant expression of RABs is involved in the dysregulation of multiple signaling pathways and many diseases such as cancer, Alzheimer’s disease, and so on [15,16,17]. In recent decades, many studies have documented that several RABs, such as RAB21 [18], RAB34 [19], RAB14 [20], and RAB27a [21] are associated with the progression and prognosis of GBM and other tumors. However, there are few studies about *RAB42* in cancers. It has been reported that *RAB42* is negatively associated with 5-year overall survival and shows a poorer prognosis in glioma patients [22]. *RAB42* is demonstrated to be involved in prenylation in vivo and in the cells [23]. A study has suggested that in keratinocytes, *RAB42* participates in protein degradation on melanosomes [24]. In addition, the potential effects of *RAB42* in GBM development and prognosis have been seldom investigated as far as we know.

Therefore, we herein aim to study the potential role of *RAB42* in GBM through deep mining of publicly obtained GBM data and further experimental validation in our local specimens. Our findings are expected to give more references for the impacts of aberrant *RAB42* in the progression of GBM.

## 2. Materials and Methods

### 2.1. Data Resources

The mRNA expression data and corresponding clinical information of 161 GBM patients were downloaded from The Cancer Genome Atlas (TCGA, https://tcga-data.nci.nih.gov/tcga/, accessed on 21 June 2020) database, among which 160 patients had complete survival information. The clinical information data of 160 patients are listed in Table 1. 

Additionally, independent mRNA expression data and corresponding clinical information of 301 GBM patients were downloaded from the Chinese Glioma Genome Atlas (CGGA, http://www.cgga.org.cn/, accessed on 21 June 2020) database, among which 285 patients had complete survival information (Table 2). Whole genome expressions of these samples were detected by the Agilent-014850 Whole Human Genome Microarray 4 × 44 K G4112F platform.

### 2.2. Clinical Samples Collection

The clinical samples were all collected from the Tianjin Huanhu Hospital (Tianjin, China), including 15 GBM tissue samples and 5 brain tissue samples. All experiments have been approved by the Ethics Committee of Tianjin Huanhu Hospital (Tianjin, China), in line with the “Declaration of Helsinki”. Informed consent was obtained from all participants. The detailed patient information of the subjects is listed in Table S1.

### 2.3. Survival Analysis

The OS of GBM patients was evaluated in the survival and survminer package of R (https://CRAN.R-project.org/package=survminer, accessed on 5 August 2020) (the difference significance was tested by log-rank).

### 2.4. Differentially Expressed Genes

Differentially expressed genes (DEGs) were analyzed in limma of R [25]. Only those DEGs with │Log_2_FC│ > 1 and FDR ≤ 0.05 were considered significant.

### 2.5. GO and KEGG Enrichment Analysis 

Regarding the DEGs, the GO (including Biological Process, Molecular Function, and Cellular Component) and KEGG pathway enrichment analysis was conducted in ClusterProfiler of R [26]. The terms with *p* value.adjust *p* < 0.05 were considered significant terms.

### 2.6. Gene Set Enrichment Analysis (GSEA) 

GSEA analysis was conducted based on the gene set c2.cp.kegg.v7.0.symbols in the MSigDB database (https://www.gsea-msigdb.org/gsea/msigdb/index.jsp, accessed on 25 November 2020) (software version: 4.0, screening criteria: *p* < 0.05).

### 2.7. Statistical Analysis

The Wilcoxon rank-sum test or Kruskal–Wallis test were employed to determine the difference significance. Multivariate Cox regression analysis was performed to find the independent OS-related factors. The statistically significant criterion was *p* < 0.05. All statistical analyses were performed with R package v3.5.2. (R Core Team, Vienna, Austria).

### 2.8. Cell Culture

In our present research, a total of four cell lines were included, comprising 3 GBM cell lines (U87, TJ905, H4) and 1 human normal astrocyte cell line, HA1800. Additionally, HA1800, TJ905, and H4 were purchased from the National Laboratory Cell Resource Platform (Beijing, China), and U87 cells were purchased from ATCC. The cell line authentication was conducted using STR profiling. All cell lines were cultured in DMEM (C11995500BT, Gibco), supplementing 100 μL/mL penicillin, 100 μL/mL streptomycin, 10% fetal bovine serum (PS, 15,140,122, Gibco), in an incubator with 5% CO_2_ at 37 °C. All cells were cultured in T25 plates at a density of 1 × 10^5^ cells per well. 

### 2.9. qRT-PCR and Reagents

The total RNA extraction was undertaken with TriQuick Reagent (Solarbio, R1100, Beijing, China), and the total RNA was subjected to concentration and purity detection. PrimeScript RT Master Mix (TaKaRa, RR037A, Beijing, China) was used for reverse transcription. Then, the amplification was conducted in the Roche 480 real-time PCR system, using SYBR Green Master Mix (ROCHE, Basel, Switzerland). The following program of PCR was used (3 repeats per sample): pre-denaturation 95 °C for 5 min, 45 cycles of 95 °C for 10 s, 60 °C for 20 s, 72 °C for 30 s. The β-actin was used for internal reference, and for all primer sequences, see Table 3. The mRNA expression was calculated by the formula 2^−^^△△CT^ [27].

### 2.10. Western Blot

All Western blot steps were in line with previous methods [28], and all proteins were assessed sequentially on the same membranes. The reagents used in our study included primary antibody *RAB42* AnTibody (H00115273-M03, 1:500, Novus, Beijing, China), secondary antibody IgG-HRP (1:10,000, Santa Cruz, CA, USA), and internal reference β-actin. The chemiluminescent HRP Substrate (BeyoECL Moon, Beyotime, Shanghai, China) was used for signal detection, and the signal was exposed with Alliance Mini HD6 (UVItec Limited, Cambridge, UK). All antibodies underwent genetic validation to verify their specificity [29]. The gray value was analyzed in software Image J and then standardized.

### 2.11. Immunohistochemistry (IHC)

The immunohistochemistry (IHC) was conducted using methods consistent with a previous report [30]. The primary antibody *RAB42* AnTibody (H00115273-M03, 1:500) and secondary antibody anti-rabbit poly-HRP-IgG (Santa Cruz, USA) were used in our study. A fully automatic immunohistochemical staining experiment was performed on the BOND MAX (Leica, DS9800, Wetzlar, Germany) instrument. The PRECICE 500B (UNIC, Beijing, China) was used for exposure.

## 3. Results

### 3.1. High Expression of RAB42 was Associated with the Development of GBM

**Figure 1:**
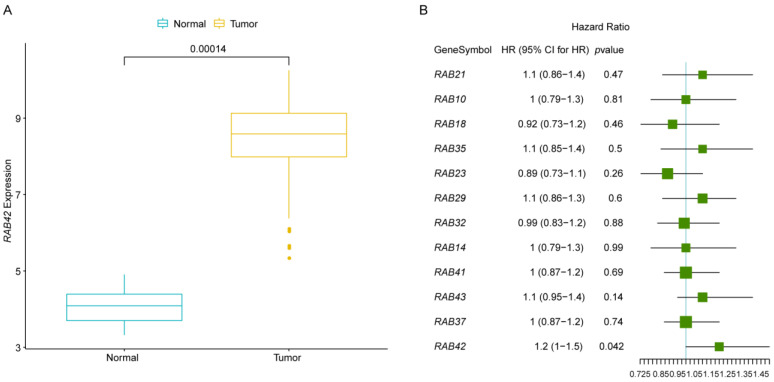
The *RAB42* expression in glioblastoma (GBM) samples and normal samples. (**A**) The expression of *RAB42* in GBM samples and corresponding normal samples based on the TCGA dataset, showed in a box plot. (**B**) Single-factor Cox analysis results of the RAB family genes based on the TCGA dataset displayed in a forest plot. HR: Hazard ratio; 95% CI: 95% confidence interval.

Using the data in TCGA, the *RAB42* expression in GBM tumor tissues and normal tissues was compared. Compared with corresponding normal samples, there was significantly higher *RAB42* expression in GBM specimens (*p* = 0.00014) (Figure 1A). In addition, 12 RAB gene family members including *RAB42* were subject to single factor Cox analysis. Our results suggested that *RAB42* was significantly correlated with the prognosis of GBM (*p* = 0.042). Moreover, the HR (hazard ratio) value of *RAB42* was greater than 1, which indicated that enhanced *RAB42* expression would lead to a poor prognosis of GBM (Figure 1B). All results above indicated that high *RAB42* expression was probably closely related to the occurrence of GBM.

### 3.2. Correlation of RAB42 Expression with the Grade, Gender, Age, and IDH Mutant Status of GBM Patients 

**Figure 2:**
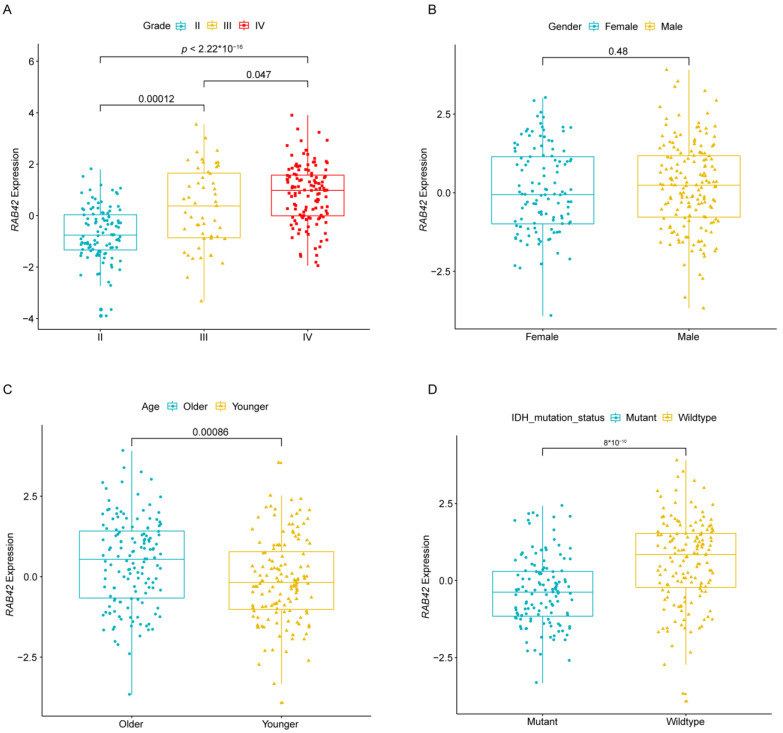
Association of *RAB42* with clinicopathological characteristics. (**A**–**D**) Based on the CGGA dataset, the correlations of *RAB42* expression with different grades, genders, ages, and *IDH* mutation status, separately.

Based on data from CGGA, the correlation of *RAB42* expression with the GBM patients’ clinicopathologic characteristics was analyzed. We found that there was a significant correlation between higher *RAB42* expression and grade (*p* < 0.05) (Figure 2A). and it was significantly enhanced along with the increase in grade (Figure 2A). In addition, no significant correlation between higher *RAB42* expression and gender was observed (Figure 2B). However, compared with younger and mutant IDH (isocitrate dehydrogenase) patients, the *RAB42* expression was significantly elevated in older and IDH wild-type GBM patients (Figure 2 C, D).

### 3.3. Highly RAB42 Expressed GBM Patients Had Worse Prognosis 

**Figure 3:**
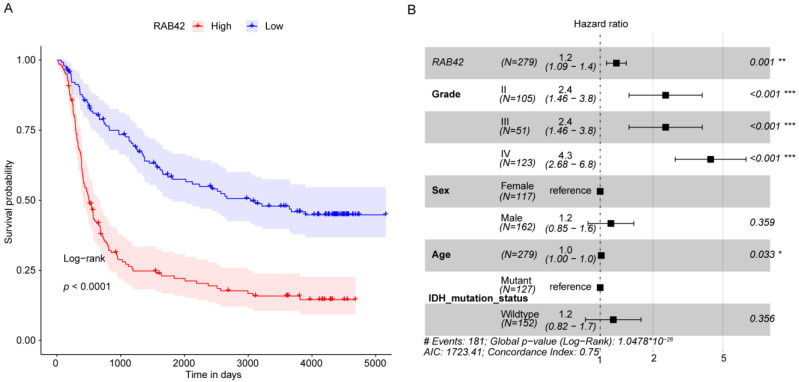
The highly *RAB42* expressed GBM patients had a worse prognosis. (**A**) Kaplan–Meier (KM) survival curve of GBM patients with high and low *RAB42* expression. (*p* determined by log-rank). (**B**) Results of the multivariate Cox regression analysis. *: *p* < 0.05, **: *p* < 0.01, ***: *p* < 0.001.

All GBM samples were divided into high and low *RAB42* expression groups based on the median *RAB42* expression. Then, a survival analysis was conducted to evaluate the prognostic value of *RAB42* in GBM data (CGGA database). Our results suggested that highly *RAB42* expressed GBM patients had poorer OS than lower *RAB42* expressed patients, which was consistent with the results obtained from the TCGA database (*p* = 2.2 × 10^−12^, HR = 0.36, 95%CI: 0.27–0.49) (Figure 3A).

Moreover, a multivariate Cox regression analysis was undertaken to find the independent prognostic indicators for GBM, comprising age, sex, grade, IDH mutation status, and *RAB42*. *RAB42* expression was still significantly associated with overall survival. High *RAB42* expression was indicated to correlate with a higher death risk (HR = 1.2, 95%CI: 1.09–1.4, *p* < 0.001) (Figure 3B).

### 3.4. Functional Enrichment Results between High and Low RAB42 Expression GBM Samples

To study the functional pathways between high and low *RAB42* expression GBM samples, we performed a differential expression analysis. Compared with highly *RAB42* expressed GBM samples, there were 675 upregulated genes and 740 downregulated genes in low *RAB42* expressed samples (Figure S1A). These 1415 DEGs then underwent a functional enrichment analysis, and they were significantly enriched in 1076 GO terms and 61 KEGG pathways. The top 20 GO terms and KEGG pathways are shown in Figure S1B,C.

### 3.5. RAB42 Expression in GBM Cell Lines and Clinical Samples

**Figure 4:**
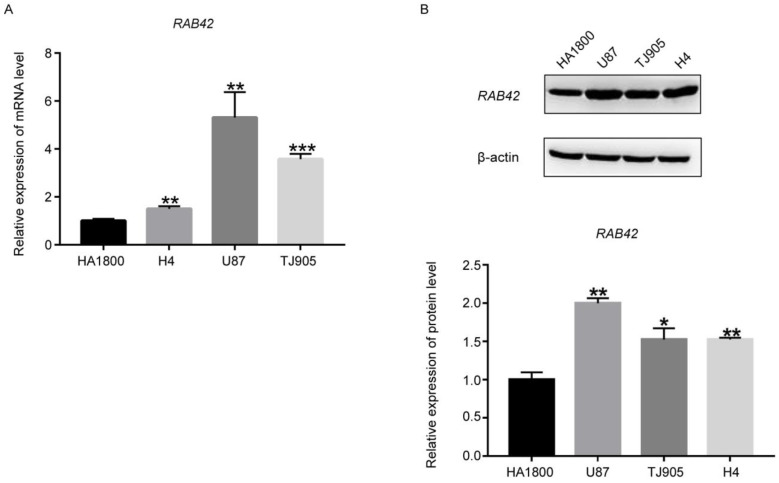
The *RAB42* expression levels in local GBM cells and specmens. (**A**,**B**) *RAB42* mRNA expression and protein expression were both upregulated in GBM cell lines, respectively (vs. HA1800; * *p* <0.05, ** *p* < 0.01, *** *p* < 0.001).

**Figure 5:**
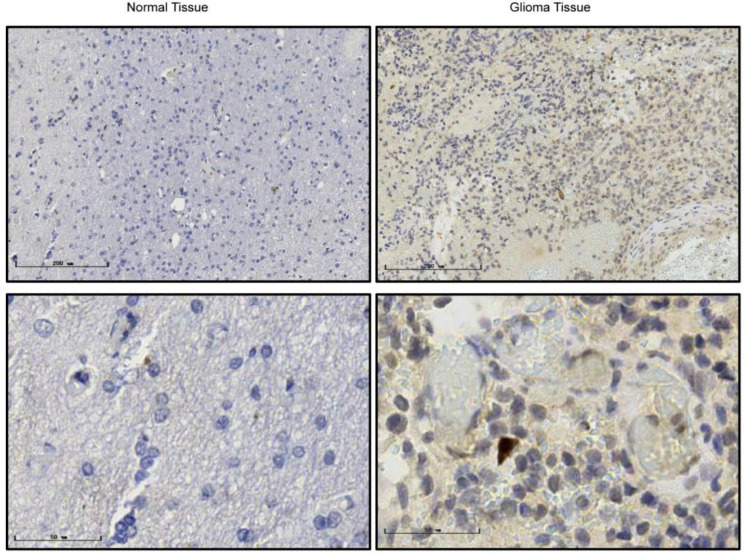
The representative results of IHC.

Based on our findings mined from the public databases, we also explored *RAB42* expression in GBM cell lines and clinical specimens. Compared with normal cell line HA1800, *RAB42* showed higher mRNA (Figure 4A) and protein expression (Figure 4B, original Western blots are shown in Figure S2) in GBM cell lines, including U87, TJ905, and H4, which were in line with our bioinformatic analysis results. Besides, our IHC results indicated that higher *RAB42* protein expression was detected in clinical GBM specimens (Figure 5), which was consistent with bioinformatic analysis.

### 3.6. GSEA Results Based on RAB42 Expression 

**Figure 6:**
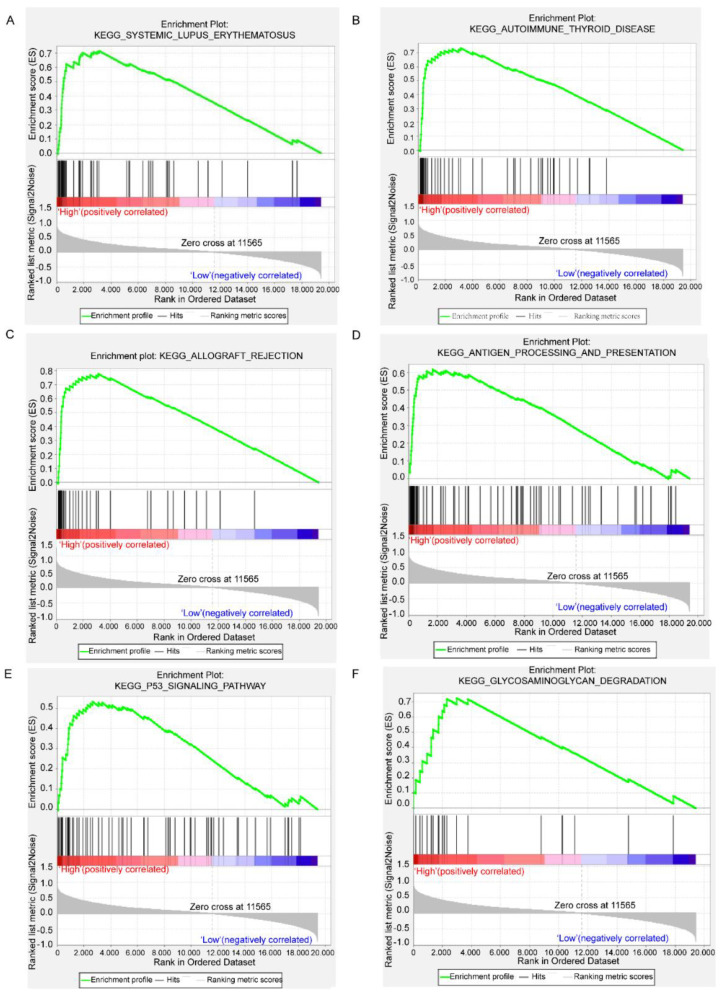
The results of GSEA enrichment of *RAB42*. (**A**–**F**) Sys-temic Lupus Erythematosus, Autoimmune Thyroid Disease, Allograft Rejection, Antigen Processing and Presentation, P53 Signaling Pathway, and Glycosamino-glycan Degradation.

Based on the data from CGGA, GSEA was performed to identify the significantly activated pathways in enhanced *RAB42* expression GBM patients compared with low *RAB42* expression GBM patients. The results revealed that 35 pathways including Systemic Lupus Erythematosus, Autoimmune Thyroid Disease, Allograft Rejection, Antigen Processing and Presentation, P53 Signaling Pathway, and Glycosaminoglycan Degradation were significantly activated (*p* < 0.05) in highly *RAB42* expressed GBM patients, compared with low *RAB42* expression samples (detailed results were listed in Table S2). According to the *p*-value, the top six significant pathways are exhibited in Figure 6.

## 4. Discussion

The malignancy and heterogeneity of GBM make it a great health burden for patients [3]. Consequently, we herein explored the correlation between *RAB42* expression and GBM based on public data in TCGA and CGGA databases. Our data implied that the high expression of *RAB42* was probably related to the development of GBM. Moreover, highly *RAB42* expressed GBM patients showed worse prognosis, indicating its prognostic biomarker probability. Additionally, enhanced *RAB42* expression was found to be significantly associated with grade, and several pathways were significantly activated in highly *RAB42* expressed GBM samples.

We demonstrated that *RAB42* was probably a promising prognostic biomarker in GBM patients. Mutant or aberrant RAB expressions were demonstrated to cause various disorders [31]. Furthermore, RABs have been reported to be up-regulated in several types of cancers [32]. *RAB42* is a member of the RAB family, while it has been hardly studied in GBM. In this study, up-regulated *RAB42* expression was observed in GBM specimens compared with normal specimens, which was successfully validated in cell lines and clinical samples. Our data indicated that high *RAB42* expression might be associated with the development of GBM. Our results partly enriched the previous similar research outcome. The research in glioma first demonstrated that *RAB42* was negatively correlated with 5-year OS and displayed a poorer prognosis [22]. In addition, it has been reported that *RAB42*, as a protein-coding gene, is related to prenylation in vivo and in cells [23], which may be indirectly involved in tumorigenesis. Not only that, another research study reported that RAB25, a member of the RAB family, exerted a promoting effect on the growth and proliferation of GBM cells [33]. A study related to RAB43, another member of the RAB family, suggested that glioma patients with high RAB43 expression showed worse clinical outcomes when compared with low RAB43 expression glioma patients [34].

Additionally, the correlation of *RAB42* with various clinicopathological characteristics and the prognosis of GBM patients was analyzed. Elevated *RAB42* expression was significantly correlated with grade. Additionally, our data indicated that the *RAB42* expression was significantly enhanced in wild-type IDH patients compared to mutant IDH GBM patients. IDH status is one of the most important genetic molecular markers of GBM [35,36], and the wild-type IDH GBMs often show poorer survival [37]. Accordingly, highly expressed *RAB42* associated with worse GBM prognosis was in line with higher *RAB42* expression in wild-type IDH GBMs. Compared with younger GBM patients, the *RAB42* expression was significantly enhanced in older patients, which was in line with a previous study showing that GBM was most commonly diagnosed in elderly patients [38]. High *RAB42* expression correlated with higher death risk, serving as a poor prognostic marker for GBM. It has been documented that various cancers are associated with upregulated RAB family members [19,33,39]. Collectively, *RAB42* is probably an independent prognostic indicator for GBM.

Moreover, *RAB42*-related pathways were identified using CGGA datasets between high and low *RAB42* expression GBM patients. Then, 35 signaling pathways were observed to be activated in high *RAB42* expression GBM patients and the six most significantly activated pathways were SYSTEMIC LUPUS ERYTHEMATOSUS, AUTOIMMUNE THYROID DISEASE, ALLOGRAFT REJECTION, ANTIGEN PROCESSING AND PRESENTATION, P53 SIGNALING PATHWAY, and GLYCOSAMINOGLYCAN DEGRADATION. We noticed that the P53 signaling pathway was significantly activated in highly *RAB42* expressed GBM patients. It has been documented that one or more genetic aberrations in the p53 pathway were contained in most GBMs [40,41,42], indicating our data were consistent with previous reports. Whether high *RAB42* expression participates in the tumorigenesis of GBM through the P53 signaling pathway and thereby negatively affects the prognosis of patients needs to be further verified. Other significantly activated pathways implied that *RAB42* exerted roles probably involving immune response (Antigen Processing and Presentation, Natural Killer Cell Mediated Cytotoxicity) and cell adhesion (Cell Adhesion Molecules CAMs, Focal Adhesion). In our research, aberrant *RAB42* expression was evidenced to activate the P53 and other signaling pathways and was related to the occurrence and prognosis of GBM. Nevertheless, there are also several limitations in this work. Although we first revealed the potential role of *RAB42* in GBM, the detailed *RAB42*-related underlying mechanisms remain unclear and deserve further investigation via wet experiments in the near future. 

## 5. Conclusions

In summary, we have revealed for the first time the potential role of *RAB42* in the development of GBM. Moreover, high *RAB42* expression might affect the prognosis of GBM by involving its progression. Highly *RAB42* expressed GBM patients’ poor prognosis indicates that *RAB42* might be a possible biomarker for GBM. All of our results may contribute to further study of the potential mechanisms in GBM and more research should be performed in the future.

## Figures and Tables

**Table 1:** Clinicopathological characteristics of GBM patients from TCGA database.

Characteristics		Patients (*n* = 160)	
		NO.	%
**Sex**	**Female**	56	35.00%
	**Male**	104	65.00%
**Age**	**≤60 (Median)**	82	51.25%
	**>60 (Median)**	78	48.75%
**Race**	**White**	143	89.38%
	**Black or African American**	11	6.88%
	**Asian**	5	3.13%
	**Unknown**	1	0.63%
**Survival Time**	**Long (>5 years)**	2	1.25%
	**Short (<5 years)**	158	98.75%
**OS status**	**Dead**	131	81.88%
	**Alive**	29	18.13%

**Table 2:** Clinicopathological characteristics of GBM patients from CGGA database.

Characteristics	*RAB42*			X-Squared	*p*-Value
		High	Low		
Age	/	45.7 ± 12.5	39.6 ± 10.2	0.43623	0.509
Sex	Female	50	67	3.1994	0.07367
	Male	88	74		
Grade	II	23	82	56.14	6.45 × 10 ^−13^
	III	27	24		
	IV	88	35		
IDH	Wild	99	53	31.435	2.06 × 10 ^−8^
	Mutation	39	88		

**Table 3:** Primer sequences for RT-PCR.

Genes	Forward Primer (5′–3′)	Reverse Primer (5′–3′)	Product Length (bp)	Tm (℃)
β-actin	CCTGGCACCCAGCACAAT	GGGCCGGACTCGTCATAC	144	60
RAB42	GGGTCATCATTAGCCCCCTT	GACCGAGTGGAAACTCCTGG	82	60

## Data Availability

The datasets generated and analysed during the current study are available in The Cancer Genome Atlas (TCGA, https://tcga-data.nci.nih.gov/tcga/, accessed on 21 June 2020) and the Chinese Glioma Genome Atlas (CGGA, http://www.cgga.org.cn/, accessed on 21 June 2020).

## Supplementary Materials

The following are available online at https://www.mdpi.com/article/10.3390/brainsci12060767/s1, Figure S1: Functional enrichment results. (A) Totally 1415 DEGs were identified. (B,C) Top 20 GO and KEGG terms, respectively; Figure S2: The original Western blots; Table S1: The clinical information of patients; Table S2: Detailed results of GSEA.
