# Supplementary material for: The Progression Related Gene RAB42 Affects the Prognosis of Glioblastoma Patients
Source: Brain Sci. 2022 Jun 11;12(6):767. doi: 10.3390/brainsci12060767 (PMC9220890; doi:10.3390/brainsci12060767)
Supplement: Supplementary file 1 [file brainsci-12-00767-s001.zip › Figure S2-v1.pdf]

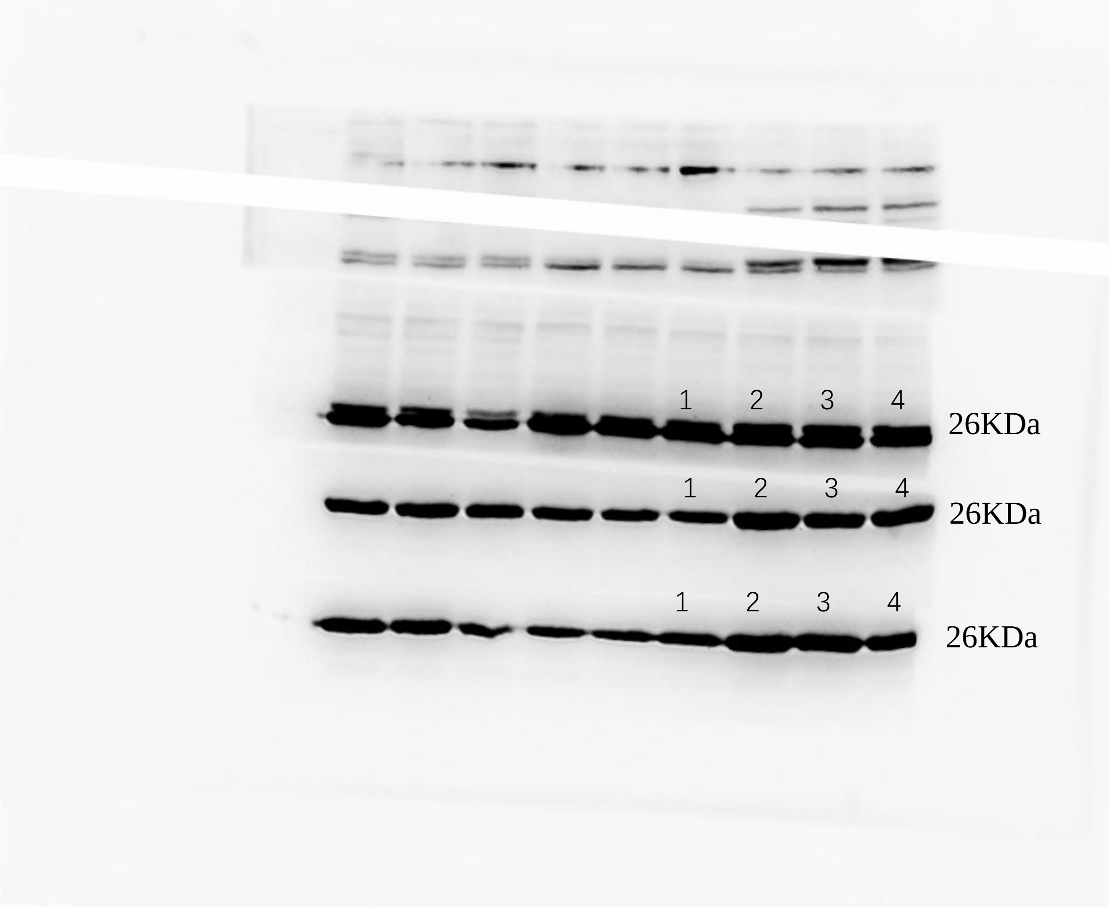

1 to 4 are HA1800, U87, TJ905 and H4 cells respectively, and the target protein is RAB42. A total of 3 repetitions were performed to obtain the optimal band.

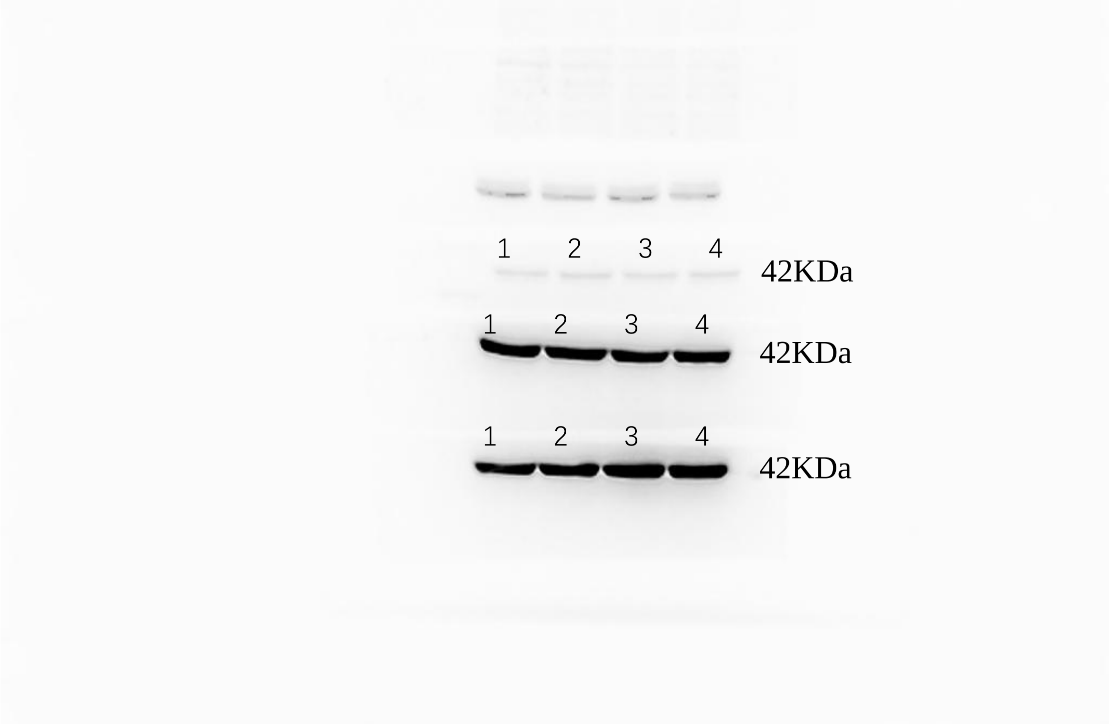

1 to 4 are HA1800, U87, TJ905 and H4 cells respectively, and the target protein is β-actin. A total of 3 repetitions were performed to obtain the optimal band.
