# Supplementary material for: The Progression Related Gene RAB42 Affects the Prognosis of Glioblastoma Patients
Source: Brain Sci. 2022 Jun 11;12(6):767. doi: 10.3390/brainsci12060767 (PMC9220890; doi:10.3390/brainsci12060767)
Supplement: Supplementary file 1 [file brainsci-12-00767-s001.zip › Table S1.pdf]

Table S1. The clinical information of patients.

No.	Pathological Grade	Age	Gender
1	II	38	Female
2	II	21	Female
3	II	49	Female
4	II	51	Male
5	IV	37	Male
6	III	84	Male
7	III	38	Male
8	II	49	Male
9	II	61	Female
10	IV	40	Female
11	II	42	Male
12	III	48	Female
13	III	36	Male
14	III	58	Male
15	II	38	Female
