# Supplementary figures and images for: The Progression Related Gene RAB42 Affects the Prognosis of Glioblastoma Patients
Source: Brain Sci. 2022 Jun 11;12(6):767. doi: 10.3390/brainsci12060767 (PMC9220890; doi:10.3390/brainsci12060767)

A

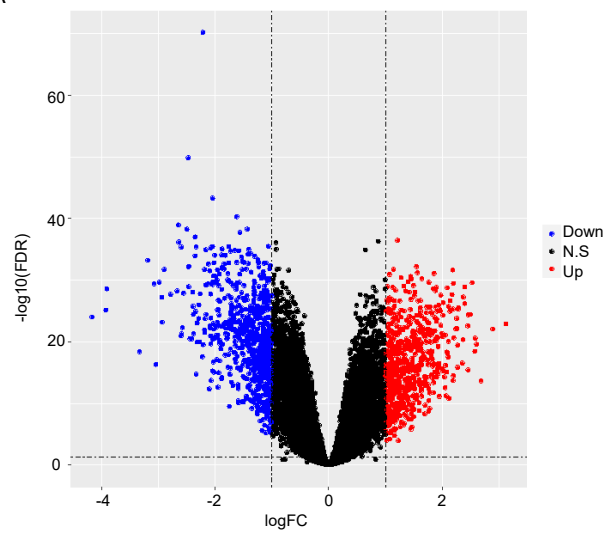

B

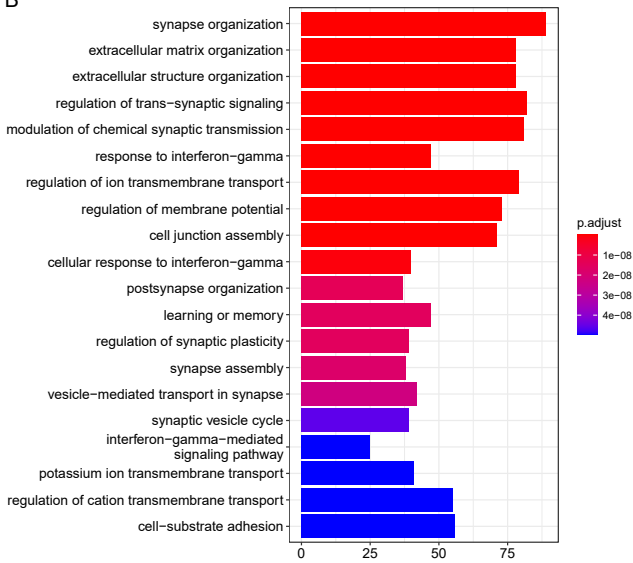

C

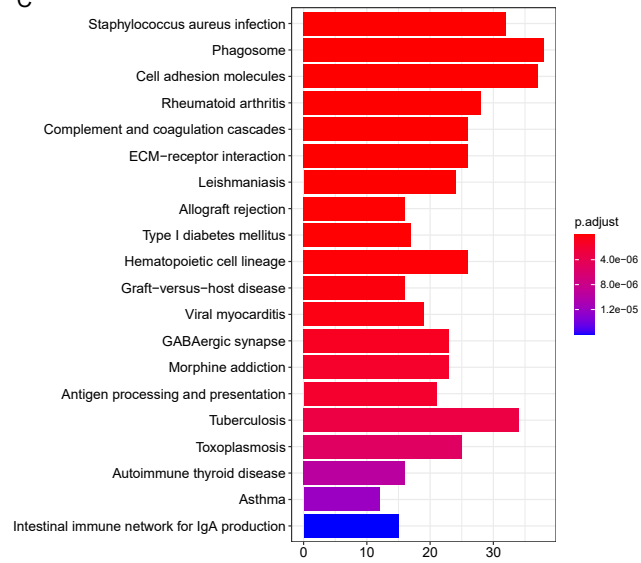

Supplement: Supplementary file 1 [file brainsci-12-00767-s001.zip › Figure S1-v1.pdf]
